# Effects of Temperature and Salt Stress on *Cereus fernambucensis* Seed Germination

**DOI:** 10.3390/biology14040393

**Published:** 2025-04-09

**Authors:** João Henrique Constantino Sales Silva, Caroline Marques Rodrigues, Aline das Graças Souza, Naysa Flávia Ferreira do Nascimento, Edna Ursulino Alves

**Affiliations:** 1 Postgraduate Program in Agronomy, Federal University of Paraíba, University Campus II, Areia 58397-000, PB, Brazil; marxcarol48@hotmail.com (C.M.R.); naysa.flavia@academico.ufpb.br (N.F.F.d.N.); ursulinoalves@hotmail.com (E.U.A.); 2 Department of Plant Science and Environmental Sciences, Center for Agrarian Sciences, Federal University of Paraíba, University Campus II, Areia 58397-000, PB, Brazil

**Keywords:** abiotic factors, beach mandacaru, coastal forest, endemism, halotolerant species, physiological mechanisms, salinity, vigor

## Abstract

The germination characteristics of cactus seeds adapted to coastal environments are crucial for understanding how climate change, such as drought and heat waves, can impact their survival and the dynamics of plant populations. This study aimed to evaluate the combined effect of temperature and salt stress on the germination and vigor of *Cereus fernambucensis* Lem. (Cactaceae) seeds and seedlings, a species endemic to Brazil. To simulate salt stress, NaCl solutions were used at the following osmotic potentials: 0.0 (control), −0.2, −0.4, −0.6 and −0.8 MPa. The seeds were kept in germinators at constant temperatures of 25, 30 and 35 °C, and evaluated daily for 21 days after sowing. The design was entirely randomized following a 3 × 5 factorial arrangement (temperature × osmotic potential). Reducing osmotic potential negatively affects the viability and vigor of *C. fernambucensis* seeds, whereas high temperatures decrease tolerance to salt stress during germination. Although these seeds can germinate in saline soils with low humidity, they are sensitive to high temperatures. Thermoinhibition was detected in the seeds at 35 °C at osmotic potentials of −0.6 and −0.8 MPa. This could limit their expansion in areas with less rainfall and affect their performance in the face of climate change, which could increase temperatures and evapotranspiration.

## Simple Summary

Beach mandacaru (*Cereus fernambucensis*) is a columnar cactus that occurs naturally in coastal vegetation on Brazilian beaches. This species produces white nocturnal flowers and fleshy fruits with several small seeds, serving as food for local fauna. This study explores the consequences of the combined effect of temperature and salt stress on the seed germination and seedling performance of this species. Our hypothesis is that increased temperature can reduce tolerance to salt stress in germinating seeds, impairing the absorption of water and nutrients essential for the initial development of seedlings. Our results show that the reduction in water availability caused by salinity negatively affects the physiological viability and vigor of *C. fernambucensis* seeds and seedlings, while high temperatures decrease tolerance to salt stress during germination. Although these seeds can germinate in soils with low or moderate salinity, they are sensitive to high temperatures. These results could improve our understanding of seed germination in saline soils in the face of climate change.

## 1. Introduction

The Brazilian Atlantic Forest is recognized as a biodiversity hotspot with high levels of Cactaceae endemism, attracting significant attention for conservation efforts [1]. Although this ecosystem is characterized mainly by its tropical evergreen forest, it also encompasses zones of xeric vegetation, such as the restinga forests found along sandy coastal plains [2]. Restinga forests are among the most threatened coastal ecosystems associated with the Brazilian Atlantic Forest [3]. Climate change is having a negative impact on biodiversity worldwide, especially in South America, where tropical forests face adverse conditions such as high temperatures and water scarcity, increasing the risk of species extinction [4,5]. Future climate projections indicate that *restinga* forests could suffer a 19% loss of species by 2050, highlighting a critical loss of biodiversity that is already underway [6].

Climate events threaten the survival of key species, such as cacti, by shaping their diversity and distribution in different ecosystems and phylogenetic contexts [7,8,9,10,11,12,13,14], especially those species that occur in coastal environments [15]. Climate change is known to modify geographical ranges, phenologies and biological interactions between species [7], but its effects on seed germination are still poorly understood. The species *Cereus fernambucensis* Lem. (Cactoideae: Cereeae) is an important component of *restinga* forests, with a natural distribution in northeastern and southeastern Brazil. Its wide distribution in open landscapes, such as coastal plains, dunes and rocky beaches, makes it a suitable model for studies on the influence of abiotic factors (simulating climate change) on seed germination and initial seedling growth.

After dispersal, the seeds of *C. fernambucensis* are exposed to the high temperature range and salinity of the soil, influenced by the sea. Evaluating the combined effect of these factors is essential to understanding the ecophysiology of the seeds and the persistence of this species in these environments. The simultaneous effects of temperature and salinity on seed germination can be harmful, as high temperatures can increase the rate of evaporation and affect the speed of enzymatic reactions, while high salinity hinders the absorption of water and essential nutrients [16]. Together, these conditions can inhibit germination, reducing seed viability and compromising the initial development of seedlings [17]. In addition, salt stress can affect critical metabolic processes such as protein synthesis and cell division, leading to increased dehydration and decreased germination rates in adverse environments [18].

Cactus seeds from northeastern Brazil have optimal germination rates between 25 and 30 °C, but temperatures above 35 °C or below 15 °C inhibit this process [19,20]. Some studies have focused their efforts on assessing the possible impact of global warming on cactus seed germination by simultaneously evaluating the effects of temperature and water potential on seed germination response [21,22,23,24,25,26]. In general, these studies showed that high germination rates were obtained by combining temperatures of 25 °C, and moist substrates of 0 MPa to −0.4 MPa. Although other studies do not directly mention global warming, they provide useful results for assessing the response of cactus seeds to temperatures above 30 °C [19,27].

The aim of this study was to evaluate the combined effect of different temperature regimes and the osmotic potential of salt solutions (NaCl) on the germination and vigor of the seeds and seedlings of *Cereus fernambucensis*, a cactus endemic to the Brazilian coast. This research arises from the hypothesis that if climate change predictions of increased temperature and decreased precipitation for tropical forests occur, there may be a reduction in the seed germination and seedling establishment of *C. fernambucensis*.

## 2. Materials and Methods

### 2.1. Fruit Collection and Seed Extraction Site

The *Cereus fernambucensis* seeds used in the experiment were obtained from mature fruits (magenta color) collected from at least 20 individuals in natural populations (7°31′41″ S, 34°49′18.3″ W) in the municipality of Pitimbu, Paraíba, Brazil. The climate in the region is tropical, with maximum and minimum temperatures of 31 °C and 21 °C, respectively, with a rainy season that begins between February and March and lasts until August, with an average annual rainfall of approximately 2100 mm [28] and relative humidity ranging from 68 to 85%. In the region, there are remnants of Brazilian coastal or *restinga* forests, environments found in the Atlantic Forest that have been largely characterized owing to their centuries-old use for a wide variety of activities [29]. The region’s soils are sandy and herbaceous vegetation and coconut groves are predominant.

During fruit collection, the soil surface temperature was measured in the population using an Instrutherm^®^—TE-500 digital skewer thermometer (Instrutherm Instrumentos de Medição Ltda, São Paulo, Brazil). The average temperature (minimum and maximum) recorded was 29.8 °C (25.5–33.5 °C).

After harvesting, the fruits were taken to the Seed Analysis Laboratory (LAS) of the Department of Plant Science of the Agricultural Sciences Center of the Federal University of Paraíba (DFCA-CCA/UFPB), where they were processed. The seeds were separated by dissecting the ripe fruit, washed with tap water to remove the mucilage via a fine mesh sieve and dried on paper towels. The seeds were placed in paper bags and stored at room temperature (24 ± 2 °C; 65 ± 5% relative humidity) for one month until the start of the germination experiment described below.

### 2.2. Setting Up and Conducting the Experiment

To carry out the germination test, the *C. fernambucensis* seeds were first disinfected with a 5% sodium hypochlorite solution (commercial bleach) for five minutes and then washed twice in distilled water to remove any residue of the disinfectant. To simulate salt stress, sodium chloride (NaCl) salts were used, the solutions of which were prepared at the following osmotic potentials: 0.0 (distilled water), −0.2, −0.4, −0.6 and −0.8 MPa (equivalent to 0, 40, 80, 120 and 160 mM NaCl, respectively), according to the Van’t Hoff equation [30]. The seeds were allowed to germinate in transparent acrylic boxes (11 × 11 × 3.5 cm). Blotting paper, previously sterilized, was used as a substrate. The paper was moistened with salt solutions at a rate of 2.5 times its dry mass, with two leaves at the base, and kept in biochemical oxygen demand (BOD) germination chambers at constant temperatures of 25, 30 and 35 °C with a 12 h photoperiod. The acrylic boxes were covered with plastic wrap to retain the humidity inside.

The number of germinated seeds was counted daily for 21 days, a period during which germination is fully stabilized, with the criterion for germination being the emergence of a radicle. Root protrusion was defined as the moment when the tip of the radicle emerged ≥1 mm through the opening of the operculum. The variables analyzed were seed water content (%), germination percentage, germination speed index [GSI = Σ(N*_t_*/D*_t_*), where N*_t_* is the number of seeds germinated on day *t*; D*_t_* is the day on which the seeds germinated; the sum was made over the total observation period] [31], mean germination time [MGT = Σ*n_i_*.*t_i_*/Σ*n_i_*, where *t_i_* is the period from the beginning of the experiment to the first observation (days) and *n_i_* is the number of seeds germinated at time *i* (number corresponding to the umpteenth observation)] [32], seedling length (cm), seedling area (cm^2^), seedling fresh mass and dry mass (mg), and the vigor index (VI = germination percentage × seedling length) [33]. At the end of the experiment, digital photographs of the seedlings were taken, and the seedling length and area were measured via ImageJ^®^ software (vesrion 1.54i). To determine the water content of the seeds, the oven method was used at 105 ± 2 °C for 24 h, in accordance with the Rules for Seed Analysis [34].

### 2.3. Design and Statistical Analysis

The statistical design used was completely randomized (DIC) following a 3 × 5 factorial arrangement (temperature × osmotic potential), with four replicates of 50 seeds (*n* = 200 seeds per treatment). The data were subjected to tests of normality and homoscedasticity of the residual variances, and then an analysis of variance (ANOVA) was carried out, with subsequent grouping of the means using the Tukey test (*p* ≤ 0.05). When significant, the quantitative data were subjected to regression analysis as a function of the osmotic potential.

The germination and vigor parameters of the seeds and seedlings were subjected to multivariate analysis. A dissimilarity measure based on the generalized Mahalanobis distance (D^2^) [35] was used. On the basis of this distance, the criterion proposed by Singh [36] was used to quantify the relative contribution of the characters to variability. Next, canonical discriminant variables were constructed to reduce the dimensionality of the data [37], which are shown in a three-dimensional scatter plot. On the basis of the same distance, to identify the existing divergence between treatments, they were grouped via the Tocher optimization method.

Statistical analyses were carried out via Genes software (Version 1990.2023.15) [38] and R version 4.2.1 [39].

## 3. Results

According to the analysis of variance (Table 1), there was a significant effect of the interaction between factors for all the characteristics evaluated (*p* ≤ 0.001), as well as for the isolated factors, i.e., osmotic potential (Op) and temperature (T) (*p* ≤ 0.001).

The water content of *Cereus fernambucensis* seeds before the experiment was conducted was 10.3%.

**Figure 1:**
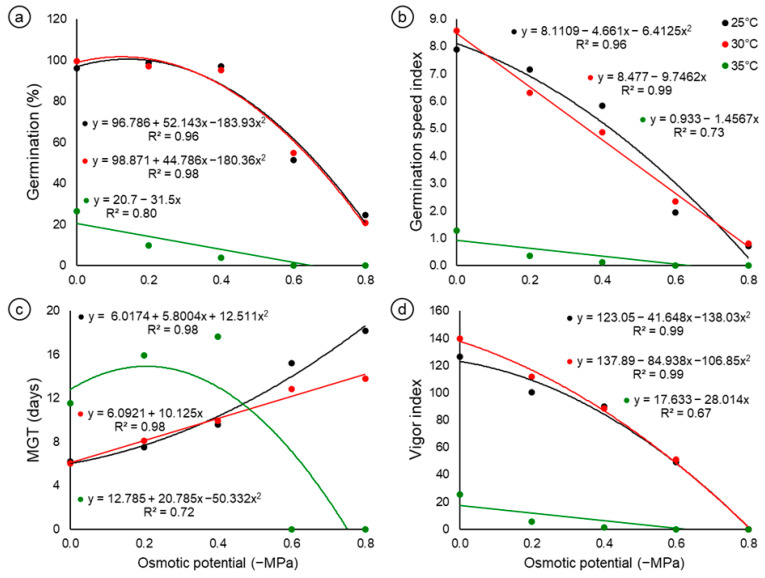
Average values of the physiological characteristics of *Cereus fernambucensis* Lem. (Cactaceae) seeds subjected to different osmotic potentials (−MPa) induced with NaCl at temperatures of 25, 30 and 35 °C. (**a**) Germination percentage; (**b**) germination speed index; (**c**) mean germination time (days); (**d**) seed vigor index.

The seeds of *C*. *fernambucensis* presented high germination percentages of up to −0.4 MPa (≥95%) when subjected to salt stress at temperatures of 25 and 30 °C (Figure 1a). At −0.6 MPa, there was a decrease in germination, the percentages of which were 52 and 55% at 25 and 30 °C, respectively, culminating in 25% (25 °C) and 21% (30 °C) at the next osmotic potential of −0.8 MPa. The temperature of 35 °C affected the germination and vigor of *C*. *fernambucensis* seeds, regardless of the osmotic potential. At this temperature, the maximum germination percentage was 27% in the control treatment (0 MPa), i.e., in the absence of salt stress. In the stress treatments, germination was observed only at potentials of −0.2 and −0.4 MPa, equivalent to 10 and 4%, respectively, resulting in zero germination at the following osmotic potentials (Figure 1a).

When the germination speed index (GSI) was analyzed, the highest values were obtained at temperatures of 30 and 25 °C in the control treatment (0 MPa), at approximately 8.56 and 7.89, respectively (Figure 1b). As the osmotic potential became more negative, the average GSI values decreased, culminating in 0.80 (30 °C) and 0.70 (25 °C) at the −0.8 MPa potential. At 35 °C, the GSI values were low (≤1.28) at all osmotic potentials, and the data fit a decreasing linear regression model (Figure 1b).

For the mean germination time (MGT), the seeds of *C*. *fernambucensis* subjected to salt stress at a temperature of 25 °C ranged from 6 days (control) to 18 days (−0.8 MPa) to germinate, i.e., they took approximately three times longer than it took for germination to occur in the control treatment (Figure 1c), whereas the seeds at a temperature of 30 °C, at the same potential (−0.8 MPa), took an average of 14 days to emit the radicle, showing that under conditions of salt stress, the MGT increased, making it difficult for the seedlings to emerge. The seeds of *C*. *fernambucensis* from the control treatment at 35 °C took approximately 12 days to germinate, but as the osmotic potential decreased, there was an increase in the MGT, except for those potentials for which there was no germination (Figure 1c).

Regarding the vigor index (Figure 1d), the highest values were observed in the control treatment, with 140.0 at 30 °C and 126.4 at 25 °C. As the osmotic potentials decreased, the performance of the vigor index also decreased, reaching zero at the most negative potential of −0.8 MPa due to the absence of normal seedlings. Although performance at both temperatures showed a similar downward trend, this fact is reinforced by the fit of a quadratic polynomial regression model, indicating a sharp decline at more negative potentials. (Figure 1d). The highest vigor index obtained for the seeds at 35 °C was approximately 25.3 for the control treatment (0 MPa). This index was even lower for seeds at potentials of −0.2 and −0.4 MPa, approximately 5.4 and 1.4, of which the data fit a linear model.

**Figure 2:**
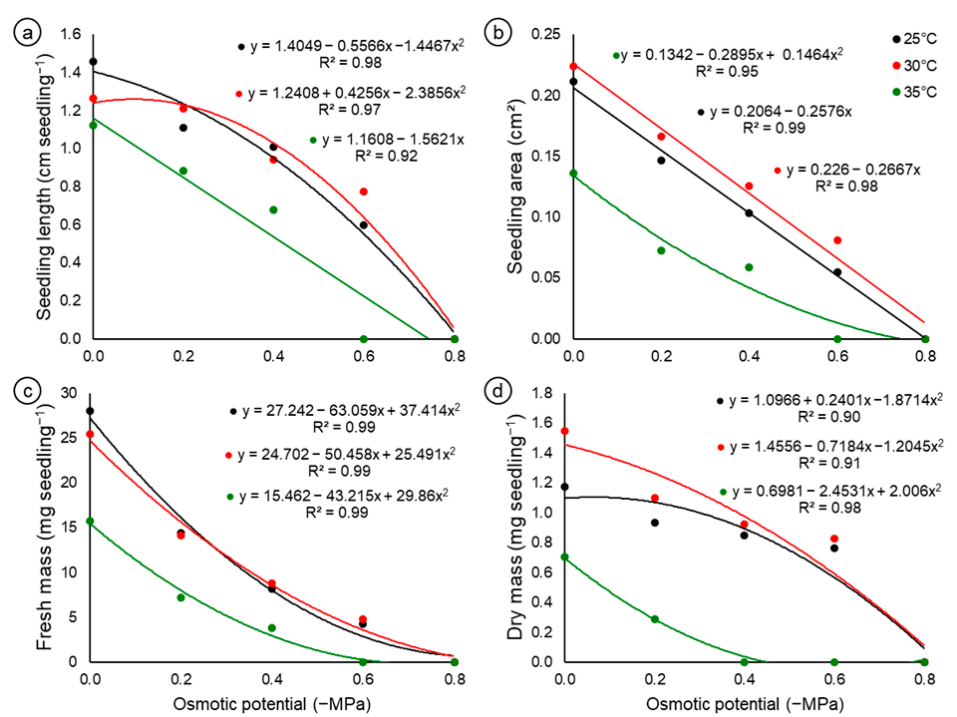
Mean values of the physical and morphological characteristics of *Cereus fernambucensis* Lem. (Cactaceae) seedlings subjected to different osmotic potentials (−MPa) induced with NaCl at temperatures of 25, 30 and 35 °C. (**a**) Seedling length (cm seedling^−1^); (**b**) seedling area (cm^2^ seedling^−1^); (**c**) seedling fresh mass (mg seedling^−1^); (**d**) seedling dry mass (mg seedling^−1^).

Compared with those in the control media, the seedling lengths in the germination media supplemented with NaCl were reduced by approximately 30.82% (25 °C), 25.98% (30 °C) and 39.28% (35 °C) at the −0.4 MPa potential (Figure 2a). This same trend was observed for the seedling area at this same potential (−0.4 MPa), where the reductions were equivalent to 52.38, 40.90 and 57.14% for the temperatures of 25, 30 and 35 °C, respectively (Figure 2b). In both situations, the size of the seedlings significantly decreased as the salt concentration increased. However, this reduction in growth was even more pronounced at the highest temperature (35 °C), indicating that the seeds and seedlings of *C*. *fernambucensis* are sensitive to high temperatures.

When the fresh mass of the seedlings was analyzed, the osmotic potential of −0.2 MPa led to a drastic reduction in this characteristic, approximately 48.61, 44.31 and 54.44% in relation to the maximum value obtained for this variable at constant temperatures of 25, 30 and 35 °C, respectively, of which the data were fitted with quadratic polynomial regression equations that explained most of the variation in the data (R^2^ = 0.99) for the three temperatures (Figure 2c). Owing to the lack of normal seedlings at more negative potentials, it was only possible to quantify the dry mass of seedlings up to −0.2 MPa (35 °C) and −0.6 MPa (25 and 30 °C) (Figure 2d). The maximum dry mass values were 1.17, 1.55 and 0.71 mg seedling^−1^ in the control treatment (0 MPa) at 25, 30 and 35 °C, respectively. The data were also fitted with quadratic regression equations. This parameter followed the same trend as the other variables mentioned above, since the average dry mass values decreased as the osmotic potentials became more negative (Figure 2d).

**Figure 3:**
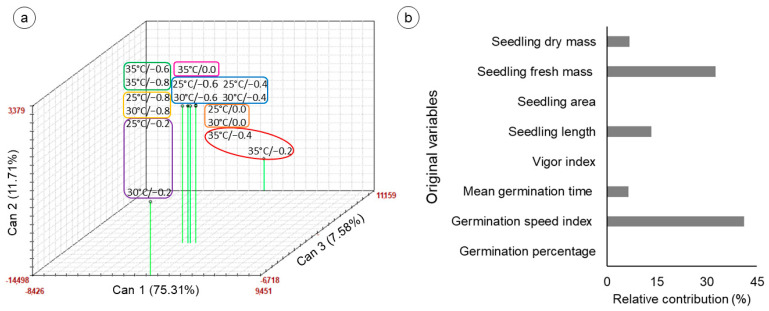
Canonical variables of original parameters in seeds and seedlings of *Cereus fernambucensis* Lem. (Cactaceae) subjected to the combined effects of different temperatures (25, 30 and 35 °C) and salinities, represented by osmotic potentials (0, −0.2, −0.4, −0.6 and −0.8 MPa) induced with NaCl. (**a**) Three-dimensional scatter diagram of the first three canonical components obtained from the germination and vigor variables of *C*. *fernambucensis* seeds and seedlings; (**b**) relative contribution of the original variables to the canonical variables, calculated according to Singh’s method. Treatments in the same rectangles or ellipses were grouped via the Tocher optimization method and the generalized Mahalanobis distance.

The first three canonical variables explained 94.60% of the variability between treatments for germination parameters and seed and seedling vigor, allowing for a three-dimensional graphical representation (Figure 3a). The treatments were separated into seven groups: group 1 (green rectangle), comprising the treatments under severe stress (35 °C at −0.6 and −0. 8 MPa); group 2 (blue rectangle), comprising the treatments at 25 °C (−0.4 and −0.6 MPa) and 30 °C (−0.4 and −0.6 MPa); group 3 (yellow rectangle), comprising the treatments at 25 and 30 °C (−0.8 MPa); group 4 (lilac rectangle), comprising the treatments at 25 and 30 °C (−0.2 MPa); group 5 (red ellipse), comprising the treatments at 35 °C (−0.2 and −0.4 MPa); group 6 (orange rectangle), comprising the treatments at 25 and 30 °C (0 MPa); and group 7 (pink rectangle), comprising the treatment at 35 °C at 0 MPa. The relative contributions of the original variables revealed that the germination speed index (41.12%) and seedling fresh mass (32.49%) were the variables that contributed the most to the total variability (Figure 3b).

## 4. Discussion

*Cereus fernambucensis* is a peculiar species of cactus that occurs in coastal ecosystems in eastern Brazil. In this study, we investigated the combined effects of temperature and salt stress on the germination and vigor of *C*. *fernambucensis* seeds and seedlings. The species response to increased temperature and salinity was negative at both the seed and seedling stages. However, *C*. *fernambucensis* also showed a relative capacity for recruitment at osmotic potentials with low water availability and mild temperatures, as discussed below.

The classification of orthodox seeds, according to Hong and Ellis [40], is based on the premise that when the water content of the seeds at the time of dispersal is less than 25%, they are probably orthodox. This was confirmed in this study, in which the water content of *C*. *fernambucensis* seeds before the experiment was set up was 10.3%. In the final phase of embryogenesis, orthodox seeds acquire tolerance to desiccation and accumulate molecules considered to be protective, such as sugars and *Late Embryogenesis Abundant* (LEA) proteins [41]. Characterizing the germination of a species provides valuable information for biology and ecology, and the parameters involved vary according to each species. These parameters include the extreme limits supported by the environment and the germination percentages observed within these limits [42].

The temperatures tested in this study are very similar to those reported in dunes under direct solar radiation in Paraiba *restinga*, where populations of *Cereus fernambucensis* occur. Given the reduction in germination at 35 °C (Figure 1a) and the high temperatures in the summer period, when fruiting and seed dispersal peak, it is expected that the seed germination of this species occurs in environments where soil moisture can be maintained for a long period of time. In the population studied, many individuals of *C*. *fernambucensis* were associated with small shrubs and coconut trees, thus suggesting a possible relationship between cactus and nurse plants. Nurse plants provide benefits during the early life cycle of protected plants, reducing the intensity of stressful abiotic conditions [43] by providing long-lasting moist microsites to meet the germination time requirements of the species.

The germination and vigor of *C*. *fernambucensis* seeds decreased as the osmotic potential became more negative. However, seeds subjected to constant temperatures of 25 and 30 °C were able to germinate at −0.4 MPa without significant losses during germination (Figure 1a). This tolerance of *C*. *fernambucensis* seeds to the stress conditions mentioned may be related to the accumulation of proteins encoded by *late embryogenesis abundant* (LEAs) genes, which are hydrophilic, heat-resistant and have no apparent catalytic activity. These proteins can interact with sugars that have the function of stabilizing membranes in stressful situations. This germination response to salt stress may explain, in addition to other factors, the wide distribution of this species in *restinga* areas, making it advantageous in these environments. According to Barrios et al. [26], cactus seeds have adapted to survive in habitats with high temperatures and low soil humidity. For most cactus species, osmotic potentials equal to or lower than −0.6 MPa considerably decrease seed germination [21,22,23,44]. Despite the decrease in germination of *C*. *fernambucensis* seeds at −0.6 MPa, germination was still higher than 50% at temperatures of 25 and 30 °C, suggesting that this species can be considered halotolerant. This fact is reinforced by the species’ natural occurrence in coastal dunes, whose soils are sandy and saline.

In a bibliographic survey carried out by Barrios et al. [20], the average germination percentage for 28 species of cacti at an osmotic potential of −0.6 MPa was approximately 26%, which was lower than that reported in the present study with *C*. *fernambucensis*, whose germination rates were 52 and 55% at temperatures of 25 and 30 °C (Figure 1a), respectively. Importantly, the aforementioned authors did not consider the temperatures tested, nor did they distinguish between the osmotic substances (mannitol, PEG and NaCl) to obtain this average, which may compromise the interpretation of the results in terms of the tolerance of the species to stress. Even so, it is interesting to note that, regardless of the osmotic agent used, at potentials of −0.6 MPa or lower, the tendency is for germination and seed vigor to be reduced for most cactus species, although some of them are considered halotolerant, as they can germinate in soils with high salinity, such as *Cereus jamacaru* DC. which can reach germination percentages close to 55% at −0.8 MPa [45].

There are reports in the literature of the significant influence of soil salinity on the germination of Cactaceae seeds, in which high levels of salts, especially sodium chloride (NaCl), can inhibit germination due to the reduction in osmotic potential, damaging the other phases of the germination process. As in the present study, the negative influence of salinity on the germination of native cactaceae seeds can also be observed in studies carried out with the species *Pilosocereus arrabidae* (Lem.) Byles and G.D. Rowley [46], *Pilosocereus catingicola* subsp. *salvadorensis* (Werderm.) Zappi [47], *Discocactus bahiensis* Britton and Rose, D. *zehntneri* subsp. *petr*-*halfarii* (Zachar) M.R. Santos and M.C. Machado, *D*. *zehntneri* Britton and Rose subsp. *zehntneri* [44], *Pilosocereus gounellei* (F.A.C.Weber) Byles and Rowley susbp. *gounellei* (=*Xiquexique gounellei* (F.A.C.Weber) Byles and Rowley susbp. *gounellei*) [48], *Cereus jamacaru* DC. subsp. *jamacaru* and *Pilosocereus pachycladus* subsp. *pernambucoensis* (Ritter) Zappi [49], of which the results revealed that the percentage of germination decreased as the concentration of NaCl in the solution increased.

When evaluating whether increasing the temperature reduces tolerance to water stress during the germination of *Pereskia grandifolia* Haw. subsp. *grandifolia* (Cactaceae) seeds, Oliveira et al. [24] noted differences in germination performance between the temperatures of 25 and 30 °C at all the potentials evaluated and reported a more drastic reduction in germination when the seeds were subjected to water deficit at 30 °C. These results are similar to those reported in the present study with the species *C*. *fernambucensis* when the seeds were subjected to 35 °C. In another study with four Cuban species of *Leptocereus*, Barrios et al. [26] reported that the negative effect of high temperature was stronger than that of reduced water potential, since at 35 °C and 0 MPa, germination was zero, except for the coastal species *Leptocereus arboreus* Britton & Rose, despite the low percentage of germination. According to these authors, the absence or low germination of *Leptocereus* spp. at 35 °C may be a response to avoid subsequent seedling mortality under high-temperature conditions.

In *Cereus uruguayanus* R. Kiesling seeds, Panetta et al. [50] reported that germination was absent at 320 mM NaCl (≅−1.6 MPa), but when the seeds from this treatment were washed and incubated again with water, germination was similar (≥91%) to that of the control, indicating that the salt did not affect the viability of the seeds. According to these authors, this suggests that the seeds are not damaged by exposure to high salinity and can persist until there is enough rain (or flooding) to reduce salinity to a level that does not prevent germination. Although the recovery of *C*. *fernambucensis* was not assessed after being subjected to the stresses in this study, it is interesting to note that a greater percentage of seed germination can occur in a shorter time after incubation in favorable conditions because the effect produced by osmoconditioning the seeds in solutions that create stress and activate events related to membrane repair and the elimination of dormancy, as observed in the seeds of *Leptocereus ekmanii* (Werd.) Knuth [26]. Additionally, according to Panetta et al. [50], the seeds of *C*. *uruguayanus* are considered tolerant to salinity, as they can germinate at 160 mM NaCl (≅−0.8 MPa) in the range of 26–81%, depending on the access tested.

In cactus species from the Chihuahuan Desert, such as *Isolatocereus dumortieri*, Flores et al. [22] reported that seeds at temperatures of 25, 32 and 36 °C, and a water potential of −0.2 MPa presented a relatively high germination percentage, which indicates that this species could germinate in a global change scenario involving high temperatures and soils that are not completely humid. The seeds of *Echinocactus platyacanthus* under relatively high temperatures and intermediate water potentials presented relatively high germination potential. The germination of *E. platyacanthus* was highest in the −0.4 MPa treatment at 32 °C, whereas the lowest germination was exhibited at 18 °C and 36 °C; however, this treatment did not require much available moisture to germinate. *Ferocactus histrix* had more seeds germinating at high moisture availability at all temperatures (18–36 °C). Bauk et al. [21], in research with populations of *Gymnocalycium monvillei* in central Argentina along an elevation gradient, reported that the combination of high temperatures and low water potentials has strong negative effects on germination: populations at higher altitudes (cooler and wetter climatic conditions) are more strongly affected by the combination of high temperature and low water potential than are populations at lower altitudes.

In another study with cacti from the Cordoba Mountains, Gurvich et al. [23] investigated the effects of a combination of temperatures (25 and 32 °C) and water potentials (0, −0.2, −0.4 and −0.6 MPa) on seed germination in six species, and reported a similar pattern between species: germination was significantly greater at 25 °C than at 32 °C, and significantly greater at 0 MPa than at lower water potentials, and no germination was recorded at −0.4 or −0.6 MPa at 32 °C. In addition, these authors reported that the lowest seedling length and width values for all the species were recorded at 25 °C/−0.2 MPa, since in the treatments with lower water potentials, the seedlings did not develop. The development of *C*. *fernambucensis* seedlings was also strongly affected by the combination of low osmotic potential and high temperature (Figure 2a–d). This effect coincides with studies by Gurvich et al. [23] for six Argentine cactus species and studies by Barrios et al. [26] for Cuban *Leptocereus* species. Parameters related to growth and biomass accumulation in Cactaceae seedlings are rarely evaluated in studies on stress, probably due to the small size of the seedlings, which limits evaluations. Additionally, these succulents are more resistant than tolerant to environmental stresses such as drought and extreme temperatures, meaning they avoid the effects of stress on their physiological processes rather than adapting through acclimation.

The simultaneous effects of temperature and water stress on the germination of two columnar cacti from a dry Brazilian tropical forest (Caatinga) were also evaluated by Silva et al. [25]. These authors applied a mathematical model to estimate the water potential that reduces the maximum germination value by 50%, and reported that the germination of *P*. *pachycladus* subsp. *pernambucoensis* seeds was reduced by 50% of its maximum value at potentials of −0.38 and −0.59 MPa at temperatures of 30 and 25 °C, respectively. For the species *C*. *jamacaru* subsp. *jamacaru*, the potentials of −0.36 and −0.40 MPa at 30 and 25 °C, respectively, reduced seed germination by 50% in relation to the maximum value. In other words, the germination response to the combined effects of temperature and water deficit varied among species and temperatures, with a temperature of 30 °C resulting in the lowest tolerance of the seeds to water stress.

**Figure 4:**
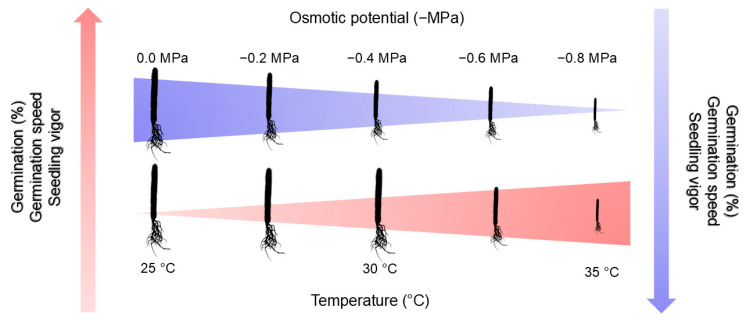
Schematic diagram of the effects of heat and salt stress, represented by different temperatures and osmotic potentials (−MPa), respectively, on the germination and initial growth of *Cereus fernambucensis* Lem. (Cactaceae) seedlings.

Rainfall at the study site where populations of *C*. *fernambucensis* occur is quite variable, and soil moisture is rarely maintained at its field capacity, either because of the texture of the soil or the high salt content. Seeds adapted to this environment can germinate in high soil humidity but below the soil’s field capacity. In general, as observed for the species studied and for many other taxa, an increase in temperature tends to reduce tolerance to osmotic stress during the germination and initial growth of cactus seedlings (Figure 4). This effect is in line with the studies by El-Keblawy and Al-Rawai [51], which indicate that the osmotic and toxic effects of sodium chloride (NaCl) can be amplified or attenuated depending on the external temperature, due to the salinity-temperature interaction. It is important to note that the cellular mechanisms that allow seeds to tolerate salinity are complex, involving molecular synthesis, enzyme induction and changes in membrane transport [52].

It is well known that tropical species tolerate high temperatures, with maximum limits of up to 35 °C or more [16]. However, in this research, the reduction in germination at 35 °C can be attributed to processes of protein thermo-denaturation, enzyme inactivation, and changes in the composition and structure of membranes, as well as the inefficiency of repair mechanisms and the synthesis of repair macromolecules, resulting in a decline in seed germination [53]. In addition, the reduction in germination and vigor of *C*. *fernambucensis* seeds can be explained by the increase in salt concentration in the germination medium, which causes a decrease in osmotic potential and a reduction in water potential, affecting the kinetics of water absorption by the seeds. In addition, high levels of toxic ion concentration in the embryo contribute to a decrease in germination potential [54].

Habitat fragmentation caused by human encroachment is one of the main factors that threatens species survival, making it crucial to understand its influence, combined with climate change, to minimize negative impacts on biodiversity and maximize species conservation [10]. Understanding the impacts of climate change on the distribution of species with ecophysiological studies is necessary to understand the tolerance of cacti to these changes in response to combinations of environmental factors [22,25], not just isolated factors (temperature or salinity). Thus, even if the species studied is not currently threatened, habitat loss due to climate change and human pressures could lead to population decline, increasing vulnerability and the risk of extinction.

The results obtained in this research reinforce the importance of understanding and adding up-to-date information on the effects of the combination of high temperatures and low water potential on the germination and initial growth of seedlings of a cactus native to Restinga forests. This knowledge is essential for the conservation of the species, as it enables more effective management strategies to be developed. We recommend selecting seeds that perform better under these adverse conditions, replanting at favorable times, monitoring environmental conditions, creating microhabitats to protect the seedlings and raising awareness about the importance of the species and the restinga forests.

The results allow us to infer the subsequent behavior of this combination under saline and thermal stress, but they do not reflect the actual tolerance of *C. fernambucensis* seeds in their natural environment. Under field conditions, under interactions between two or more abiotic factors, the seeds can experience a cascade of events that are barely perceptible or measurable in terms of their impact on seed germination in their habitat and the establishment of *C. fernambucensis* populations. Thus, studies on Cactaceae aimed at the germination process of seeds of *C. fernambucensis* occurring in the *Restinga* should be encouraged and revealed in the face of climate change and anthropogenic interventions to understand the establishment and chances of survival of these species in their natural environment. Therefore, this information should be taken into account in conservation programs to ensure the preservation and resilience of these populations at their occupation sites.

## 5. Conclusions

Reducing osmotic potential decreases the viability and vigor of *Cereus fernambucensis* seeds, whereas increasing temperature decreases tolerance to salt stress during germination. Despite this, these seeds are able to germinate in saline soils with low water availability, which is a typical characteristic of *Restinga* areas; however, they are sensitive to a temperature of 35 °C, even in the absence of salinity stress. The germination of *C*. *fernambucensis* seeds may limit their expansion to areas with lower levels of rainfall, as well as their performance in the context of climate change, where an increase in temperature is expected to lead to higher rates of evapotranspiration. Future work should explore the phenotypic plasticity of *C*. *fernambucensis* seed germination and the formation of a seed bank in the soil.

## Figures and Tables

**Table 1:** Results of the analysis of variance (ANOVA) of the physiological characteristics of the seeds and seedlings of *Cereus fernambucensis* Lem. (Cactaceae) subjected to different temperatures and osmotic potential levels induced by salt stress.

Sources of Variation	df	Mean Squares ^1^			
		GP (%)	GSI	MGT (Days)	VI
Osmotic potential (Op)	4	7687.6 ***	59.744 ***	32.761 ***	16,748 ***
Temperature (T)	2	28,514.4 ***	122.893 ***	26.911 ***	32,179 ***
Op × T	8	1080.1 ***	10.355 ***	203.027 ***	2714 ***
Residue	45	110.1	0.405	3.193	131
CV (%)		17.6	17.2	15.2	18.8
		SL (cm)	SA (cm^2^)	SFM (mg)	SDM (mg)
Osmotic potential (Op)	4	3.1373 ***	0.0652 ***	981.62 ***	2.0753 ***
Temperature (T)	2	0.6030 ***	0.0239 ***	200.51 ***	3.1082 ***
Op × T	8	0.1024 ***	0.0017 ***	23.27 ***	0.2672 ***
Residue	45	0.0117	0.0002	2.84	0.0332
CV (%)		12.7	15.7	16.2	24.3

^1^ *** significant at 0.1% probability according to the F test. Degree of freedom (GL), coefficient of variation (CV), germination percentage (GP), germination speed index (GSI); mean germination time (MGT), vigor index (IV), seedling length (SL), seedling area (SA), seedling fresh mass (SFM) and seedling dry mass (SDM).

## Data Availability

The original contributions presented in the study are included in the article. The data presented in this study are available on request from the corresponding author.
